# Accuracy of the tuberculosis point-of-care Alere determine lipoarabinomannan antigen diagnostic test using α-mannosidase treated and untreated urine in a cohort of people living with HIV in Guatemala

**DOI:** 10.1186/s12981-020-00318-8

**Published:** 2020-10-19

**Authors:** Juan Ignacio García, Johanna Meléndez, Rosa Álvarez, Carlos Mejía-Chew, Holden V. Kelley, Sabeen Sidiki, Alejandra Castillo, Claudia Mazariegos, Cesar López-Téllez, Diana Forno, Nancy Ayala, Joan-Miquel Balada-Llasat, Carlos Rodolfo Mejía-Villatoro, Shu-Hua Wang, Jordi B. Torrelles, Janet Ikeda

**Affiliations:** 1 Fundació Sida i Societat, Technical Advisor Unit (UAT), Escuintla National Hospital, Escuintla, 5001 Guatemala; 2 Population Health Program, Tuberculosis Group, Texas Biomedical Research Institute, San Antonio, TX 78227 USA; 3 Unidad de Atención Integral del VIH e Infecciones Crónicas del Hospital Roosevelt “Dr. Carlos Rodolfo Mejía Villatoro”, Guatemala City, Guatemala; 4 Sección de Microbiología, Departamento de Laboratorios Clínicos, Hospital Roosevelt, Guatemala City, Guatemala; 5 Division of Infectious Diseases, Department of Medicine, Washington University School of Medicine, St. Louis, MO 63110 USA; 6 Clinica de Atención Integral Dr. Isaac Cohen Alcahé, Hospital de Especialidad Dr. Robles, Quetzaltenango, Guatemala; 7 Asociación de Investigación, Desarrollo y Educación Integral (IDEI), Quetzaltenango, Guatemala; 8 Division of Global HIV/AIDS, Centers for Disease Control and Prevention, Central America Regional Office, Guatemala City, Guatemala; 9 National Reference Laboratory, Amatitlan, Guatemala; 10 Department of Pathology, The Ohio State University, Wexner Medical Center, Columbus, OH USA; 11 Internal Medicine Department, Infectious Disease Division College of Medicine (COM), The Ohio State University (OSU), Columbus, OH 43210 USA

**Keywords:** Tuberculosis, Lipoarabinomannan antigen, Point-of-care diagnosis, HIV, Urine, *Mycobacterium tuberculosis*

## Abstract

**Background:**

Improved point-of-care diagnostic tests for tuberculosis (TB) in severe immune suppressed people living with HIV (PLWH) are needed to decrease morbidity and mortality outcomes. The aim of the study is to evaluate the performance of the lipoarabinomannan antigen test (LAM-test) with and without α-mannosidase pre-treated urine in a cohort of PLWH in primary care clinics in Guatemala. We further determined TB incidence, and mortality rates and its risk factors in PLWH with TB symptoms.

**Methods:**

Prospective longitudinal study of PLWH with TB symptoms. Urine samples were collected at 2 HIV sites to test the sensitivity of the LAM-test in urine with and without α-mannosidase pre-treatment. A composite reference standard of either a positive *Mycobacterium tuberculosis* complex culture and/or GeneXpert^®^ MTB/RIF (Xpert, Cepheid, Sunnyvale, CA, USA) results was used in the LAM-test diagnostic accuracy studies. Cox proportional hazards regression was used to study mortality predictors.

**Results:**

The overall sensitivity of the LAM-test was of 56.1% with 95% CI of (43.3–68.3). There were no differences in the LAM-test sensitivity neither by hospital nor by CD4 T cell values. LAM-test sensitivity in PLWH with < 200 CD4 T cells/µl was of 62.2% (95% CI 46.5–76.2). There were no significant differences in sensitivity when comparing LAM-test results obtained from untreated vs. α-mannosidase treated urine [55.2% (95% CI 42.6–67.4) vs. 56.9% (95% CI 44–69.2), respectively]. TB incidence in our cohort was of 21.4/100 person years (PYs) (95% CI 16.6–27.6), and mortality rate was of 11.1/100 PYs (95% CI 8.2–15.0). Importantly, PLWH with a positive LAM-test result had an adjusted hazard ratio (aHR) of death of 1.98 (1.0–3.8) with a significant *p* value of 0.044 when compared to PLWH with a negative LAM-test result.

**Conclusions:**

In this study, α-mannosidase treatment of urine did not significantly increase the LAM-test performance, however; this needs to be further evaluated in a large-scale study due to our study limitations. Importantly, high rates of TB incidence and mortality were found, and a positive LAM-test result predicted mortality in PLWH with TB clinical symptoms.

## Introduction

*Mycobacterium tuberculosis* culture is the reference standard for tuberculosis (TB) diagnosis worldwide; however, it is not routinely used in low-income countries due to its high cost, lack of laboratory capacity and long turnaround time (2 to 6 weeks). Consequently, in high TB burden settings with limited resources, sputum smear microscopy detecting acid fast bacilli (AFB) remains the main laboratory TB diagnostic test for the majority of rural health care centers [1, 2].

In 2010, the World Health Organization (WHO) endorsed the GeneXpert^®^ MTB/RIF assay (Xpert, Cepheid, Sunnyvale, CA, USA) as the initial diagnostic test for people living with HIV (PLWH) with presumptive pulmonary TB (PTB). The Xpert rapidly (2-h) detects 99% of smear-positive and 70% of smear-negative in people with PTB [3]. Conversely, severely immune suppressed PLWH with presumptive extrapulmonary or disseminated TB fail in getting a positive Xpert result, mainly because they are too ill to produce a quality sputum sample, or their sputum contains very few AFB. For these individuals, currently the Xpert is unlikely to be useful and thus, the need of alternative TB diagnostic tests with increased sensitivity to provide early treatment and care and reduce mortality in PLWH having extrapulmonary or disseminated TB [4].

Lipoarabinomannan (LAM) is a *M. tuberculosis* cell envelope lipoglycan that is being explored as a biomarker for active TB disease. LAM is a heterogeneous, stable, immunogenic, and virulent factor thought to be released into the milieu by active or degrading bacilli [5, 6]. *M. tuberculosis* LAM is a tripartite structure composed of a lipid moiety [glycosyl-phosphatidyl-*myo*-inositol (GPI) anchor] with different degree of acylation by different fatty acids. It also contains D-Mannan and D-Arabinan domains, and in its non-reducing end has mannose-caps, defining the typical *M. tuberculosis* complex ManLAM [7]. LAM heterogeneity resides at the different degrees of acylation in its GPI-anchor, mannose-capping, and branching in both D-Mannan and D-Arabinan domains.

Once released into the bloodstream, LAM is filtered by the kidneys and detected in the urine [8, 9]. In this context, Alere Determine TB LAM Ag test (LAM-test) performed in urine has great expectations for its potential to improve the diagnosis of TB in PLWH with low CD4 cell counts, and who are at greatest risk of death if TB remains undiagnosed. There are several studies that performed LAM-test TB diagnosis accuracy assays in TB high burden areas [10]. Lawn et al. described that among PLWH eligible to start antiretroviral treatment in South Africa, the urine LAM-test has highest sensitivity for the detection of culture positive PTB at low CD4 cell counts. However, in this study, the estimates precision of the LAM-test was poor due to wide and overlapping confidence intervals, with 66.7% sensitivity (95% CI 41.0–86.7) in PLWH with CD4 < 50 cells/µl, 51.7% (32.5–70.6) at CD4 < 100 cells/µl, and 39.0% (26.5–52.6) at CD4 < 200 cells/µl [11]. Notably, this study identified a subgroup of PLWH with TB disease with poor prognosis and high mortality. Conversely, Peter et al. observed a higher sensitivity in those with CD4 < 200 cells/ml (72%, 95% CI 61–80) compared to CD4 > 200 cells/ml (54%, 95% CI 36–71) [12]. This variability observed is thought to be related to: (i) the failure to contain the infection in the lungs resulting in disseminated TB with renal involvement and presence of LAM in urine; (ii) dysfunctional humoral immunity results in abundant free LAM, which can pass into the urine through the glomerular filtration, whereas immune complexes of antibody-bound LAM do not; (iii) dysfunctional cellular immunity drives host susceptibility to less virulent mycobacteria, which may have different LAM structures; and/or iv) different geographically distributed strains presenting diverse LAM structures [13].

To date, no studies have evaluated the LAM-test in Central America. Herein, we focused on the LAM-test to address its efficacy in detecting *M. tuberculosis* in a cohort of PLWH with TB symptoms in Guatemala. We also determined if a 30 min α-mannosidase pre-treatment of urine using an enzyme that removes the mannose caps of LAM could improve the sensitivity of the LAM-test as we have shown in the laboratory settings [14], and further correlated LAM-test results with TB incidence, mortality rates, and risk factors in our cohort of PLWH.

## Materials and methods

### Ethic committee

The Ethical Review Committee of the Guatemalan Ministry of Health and Social Welfare approved this study protocol with approval number 45-2014. The Institutional Review Board at The Ohio State University approved this study protocol with approval number 2013H0251.

### Study design

A prospective cohort study of PLWH recruited from April 2015 to December 2017. Follow up period ended on December 31st, 2017. All PLWH with clinical (4-Symptoms WHO active TB screening: i.e., cough, weight loss, night sweats, and fever [15]) and/or radiological abnormalities concerning for PTB or extrapulmonary TB were offered to participate in this study. A total of 361 PLWH with TB symptoms were enrolled and followed up during the study period. After TB/HIV counselling, participants provided their written inform consent and were recruited following our human subjects IRB approved protocols. To estimate TB incidence and death rates, person-years of follow up (PYs) were calculated using participants’ entry date to the study and censoring occurred at either TB date, lost to follow up, death, or study recruitment ending date (December 31st 2017). The last study participant was recruited in December 6th 2017.

### Study settings

This study took place in two UAI clinics (‘Unidad de Atención Integral’) in Guatemala. UAI clinics function as a One Stop TB/HIV service delivery model [16] for integral management of the TB/HIV syndemic [17]. The ‘Dr. Isaac Cohen Alcahé’ UAI (henceforth UAI 1) is located within the regional, respiratory infection and TB Specialty Hospital ‘Rodolfo Robles’ in the rural highland region of Quetzaltenango, Guatemala. It attends mostly to indigenous individuals, who come from distant communities in western Guatemala and the southern part of Chiapas, Mexico. These communities have limited resources and low educational levels, and an increasing number of lesbian, gay, bisexual, and transgender (LGBT) populations [18, 19]. The other UAI involved in this study is the ‘Dr. Carlos Rodolfo Mejía-Villatoro” Clinic (henceforth UAI 2) the largest HIV clinic in Guatemala, located within the Roosevelt Hospital in Guatemala City, one of the two national reference hospitals in Guatemala. UAI 2 attends individuals that have been referred from other smaller UAIs and from patients from all over the country. Participants in this study were both, ambulatory and hospitalized patients admitted from emergency services, external consultations within hospitals and waiting rooms in UAIs. Site 1 includes UAI 1 and Hospital Rodolfo Robles and Site 2 includes UAI 2 and Roosevelt Hospital. Enrolled PLWH initiated anti-TB treatment in accordance with the National TB Control Program of Guatemala guidelines [20]; urine samples were collected from all enrolled subjects prior they started their anti-TB treatment.

### TB diagnosis

All subjects were recruited from inpatient and outpatient setting and screened for WHO-defined four TB clinical symptoms [21]. Rapid antibody HIV tests were offered to all individuals with TB clinical symptoms. Positive rapid tests were confirmed with fourth generation ELISA/P24 tests. The study team (infectious disease specialist, a nurse, and a microbiologist) explained risks and benefits of participating in the study and obtained informed consent. Potential participants were screened for inclusion criteria.

The Gold standard for TB diagnosis was a composite reference standard defined as either a *M. tuberculosis* positive culture or a positive Xpert result. Samples obtained included smear sputum for acid-fast bacilli (AFB) and Xpert, and urine for Xpert and LAM-test. In some instances, abscesses, biopsies or other body fluids were taken. Both UAI 1 and UAI 2 followed the combined antiretroviral therapy (cART) protocol for early cART initiation (2 weeks after the onset of anti-TB treatment [20]). Ambulatory PLWH were referred to the health center nearest to their residence to initiate anti-TB direct observed treatment (DOT).

### Laboratory procedures

For mycobacteria culture, clinical specimens (sputum, cerebrospinal fluid, pleural fluid, biopsy lysates) were first decontaminated using the BD BBL MycoPrep™ system. Once decontaminated, 5 drops (50 μL/drop) of the decontaminated sample were added into two tubes with Löwenstein-Jensen (LJ) medium and in UAI 2, 500 μL were added into the BD BACTEC™ MGIT (automated liquid mycobacterial growth indicator tube) system for mycobacteria growth detection. LJ tubes were incubated at 37 °C and taken out for manual reading twice a week for 8 weeks. All solid (LJ) and liquid (MGIT) cultures suggestive of *M. tuberculosis* growth were further tested with the SD BIOLINE TB Ag MPT64 immunochromatographic test to confirm *M. tuberculosis* complex strains. MPT64 test negative isolates were verified later as possible nontuberculous mycobacteria (NTM). The final identification of all *M. tuberculosis* complex strains was confirmed using HAIN LifeScience GenoType *Mycobacterium* CM and GenoType Mycobacterium AS identification kits. Drug susceptibility of the *M. tuberculosis* confirmed strains was performed using the HAIN (LifeScience GenoType MTBDRplus and GenoType MTBDRsl) kits. In UAI 1, all solid (LJ) cultures suggestive of *M. tuberculosis* growth were further tested for drug susceptibility at the National Reference Laboratory.

The Xpert test was performed from decontaminated clinical specimens. Following the manufacturer’s instructions, 1 ml of the specimen was mixed with 2 ml of the kit reagent. The mixture was pipetted into the cartridge and inserted into the Xpert instrument for processing.

All urine samples were collected at recruitment, and stored at − 20 °C before use. For each individual, the same urine sample was used to perform the LAM-test in both, untreated and α-mannosidase treated urine. Briefly, for the LAM-test, 60 μL of urine were directly pipetted on the LAM-test strip. After 25 min, results were read and interpreted following the manufacturer’s instructions [3]. For the α-mannosidase treatment of urine, a urine-sodium bicarbonate mixture (1:1, v/v, 100 μL final volume) was treated with 0.1 IU of α-mannosidase (Sigma-Aldrich, San Louis, MO) and incubated at 37 °C for 15 min. The LAM-test was performed using 60 μL of this mixture according to the manufacturer’s instructions. A LAM-test was read as positive using an intensity band grade scale from 1 to 4. Two independent clinicians agreed on readings for both, LAM-test and LAM-test after α-mannosidase treatment of urine; however, LAM-test band intensity readings were not recorded.

### Study definitions

Combinational antiretroviral therapy (cART) regimens are defined in Table 1. Diabetic PLWH were defined as those with percentage of haemoglobin A1C (% Hba1c) measurements above 6.1 [22]. Body mass index (BMI) was re-categorized as binary defining ≥ 17 kg/m^2^ and < 17 kg/m^2^. WHO HIV stage was binary defined as 1 or 2 and 3 or 4.

**Table 1:** Characteristics at baseline registration of PLWH enroled by Site

Variable			Site^a^1, n = 266		Site^a^ 2, n = 95	*p* value^b^
		n^c^	%^d^	n^c^	%^d^	
Age	Median (IQR)	264	36 (28–51)	95	36 (31–44)	0.831
Sex		266		95		0.229
	Male		71.54		77.89	
	Female		28.46		22.11	
Diabetes^e^		245		32		< 0.001
	Yes		3.27		50.00	
	No		96.73		50.00	
BMI^f^		217		95		0.146
			19.71 (17.34–22.40)		18.82 (16.60–21.50)	
TB symptoms						
Cough		264		71		0.639
	Yes		72.24		74.74	
Weight lost		265		91		NA
	Yes		48.11		100	
Night sweating		264		95		0.002
	Yes		48.11		66.32	
Fever		263		79		NA
	Yes		39.54		100	
CD4 values at baseline		247		95		< 0.001
			185.50 (89–398)		49 (19–121)	
Viral load (copies/ml)		241		95		0.059
			39 200 (861–173 000)		93 616 (6044–242 469)	
cART regimen^g^		226		95		< 0.001
	1st line		89.38		34.74	
	2nd line		10.62		65.26	
cART defaulters						
	Yes		18.72		18.00	0.431
Death		260		95		
	Yes		11.20		27.37	< 0.001

*NA* Not applied

^a^Site = Site 1 includes UAI 1 = “Dr. Isaac Cohen Alcahé” UAI and Rodolfo Robles Hospital. Site 2 includes UAI 2 = “Dr. Carlos Rodolfo Mejía-Villatoro” UAI and Roosevelt Hospital

^b^Pearson’s Chi squared p values for categorical variables and Mann-Whitney median comparison p value for independent samples

^c^Variable sample size

^d^Data are number (%) or median (IQR)

^e^Hba1c measurements in % above 6.15 were considered the threshold for diabetic patients

^f^In kg/m^2^

^g^1st line regimens are based on a-ABC in combination with 3TC and either EFV or NVP or LPV/RTV, or ABC in combination with AZT and either EFV or LPV/RTV, or ABC in combination with TDF and EFV; b-AZT in combination with 3TC and either EFV, or NVP, or LPV/RTV or ABC; c-TDF in combination with FTC and either 3TC, or EFV, or NVP, or LPV/RTV, or AZT and 3TC

2nd line regimens are based on: a-ABC in combination with AZT and LPV/RTV, or in combination with DDI plus EFV, or DDI plus NVP or DDI plus SQV/RTV, b-AZT in combination with 3TC plus ATV, or ATV/RTV or ATV/SQV, or AZT in combination with DDI plus LPV/RTV, c-DDI in combination TDF and EFV, or DDI in combination with 3TC plus NVP, or EFV, or SQV/RTV, or LPV/RTV

3rd line regimens and regimens including RAL, DRV, MRV used due to genetic resistance are included in 2nd line regimens. AZT (azidovudine), FTC (emtricitabine), TDF (tenofovir), NVP (nevirapine), ABC (abacabir), 3TC (lamivudine), LPV/RTV (lopinavir/ritonavir), EFV (efavirenz), ATV (atazanavir), RAL (raltegravir), DRV (darunavir), MRV (maraviroc), DDI (didanosine)

### Statistical analysis

In the descriptive analysis, proportions were compared using the Chi square test. U Mann–Whitney was used to compare medians in different groups as appropriate. The significance for all comparison tests was set at *p *< 0.05. Sensitivity and specificity were compared between the two participating UAIs using the Fischer exact test. Cox proportional hazard regression was used to calculate unadjusted (uHR) and adjusted hazard (aHR) ratios to explore predictors of mortality in the cohort of PLWH with TB symptoms. The following confounding factors were used for adjustment: age, sex, CD4 values, cART, viral load, and WHO HIV stage. Data analyses were conducted using Stata, ver. 14 (Stata Corporation, College Station, TX, USA).

## Results

### Demographic and clinical characteristics of the PLWH enrolled

**Fig. 1:**
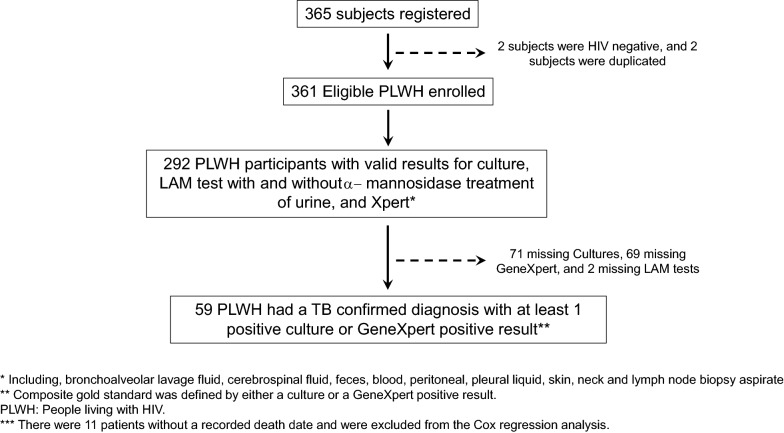
Patient flowchart for analyses of LAM and α-mannosidase urine LAM-test sensitivity and specificity and for mortality predictors

PLWH flow charts for the diagnostic accuracy analysis of the LAM-test vs. our composite referenced standard, and for the LAM-test performed in untreated vs. α-mannosidase treated urine are depicted in Fig. 1. Briefly, 292 out of 361 (80.9%) eligible PLWH had valid results for Xpert, culture and both LAM-tests (untreated *vs.* α-mannosidase treated urine) and thus, these were used in the diagnostic accuracy analysis. PTB disease was diagnosed in 59/292 (20.2%) PLWH with TB clinical symptom/radiological abnormality. Finally, there were 343 subjects with TB symptoms with information on mortality outcomes that were used to study predictors of death by Cox regression analysis.

Enrolled PLWH characteristics at baseline are shown in Table 1. No differences were observed in age, sex and BMI at both study sites, except for clinical symptoms of weight loss and fever. Interestingly, Site 2 had higher proportion of second line cART regimens (65.3% vs. 10.6%) and higher mortality risk (27.34% vs. 11.2%) with *p* values < 0.001 when compared to Site 1.

### Diagnostic accuracy of the LAM-test vs. the composite gold standard by Site and CD4 values

We first determined the diagnostic accuracy analysis of the LAM-test compared to the defined composite referenced standard. Overall, LAM test sensitivity was of 56.1 with 95% CI (43.3–68.3), and 62.2% with 95% CI (46.5–76.2) in PLWH with CD4 T cell values < 200 cells/µl. There were no differences in the LAM-test sensitivity neither by UIA/Sites nor by CD4 T cell values.

### Sensitivity and specificity comparison analysis of LAM-test in untreated vs. α-mannosidase treated urine using the composite reference standard stratified by CD4 T cell values and Site

We further determined the sensitivity and specificity of the LAM-test in α-mannosidase treated vs. untreated urine when compared to the defined gold standard composite. Results were stratified by Site and CD4 T cell values (Table 2). No differences were observed in the performance of the LAM-test with and without α-mannosidase treatment of urine, neither when stratified by Site nor by CD4 values. There were 3 PLWH that had a positive LAM-test result only when their urine was α-mannosidase treated. Only 1 out of these 3 PLWH also had a positive culture. Conversely, there were 5 PLWH that their LAM-test result converted from a positive to a negative when their urine was α-mannosidase treated. Only 1 out of these 5 PLWH also had a confirmed TB positive culture, the others presented negative cultures.

**Table 2:** Sensitivity and specificity comparisons of LAM test after α-mannosidase treatment of urine vs. LAM test by Site and CD4 values using a composite gold standard

	LAM after α-mannosidase treatment of urine		LAM alone		*p* value^b^	LAM after α-mannosidase treatment of urine		LAM alone		*p* value^b^
All CD4 values	Sensitivity (% (95% CI)		Sensitivity (% (95% CI)			Specificity (% (95% CI)		Specificity (% (95% CI)		
	Positive/N		Positive/N			Negative/N		Negative/N		
Both Sites^a^	37/59	56.9 (44–69.2)	37/59	55.2 (42.6–67.4)	1	204/232	90.3 (85.6–93.8)	202/232	90.2 (85.5–93.7)	0.946
Site1	26/41	52 (37.4–66.3)	26/41	49.1 (35.1–63.2)	1	194/218	92.8 (88.4–95.9)	191/218	92.7 (88.3–95.9)	0.944
Site 2	11/18	73.3 (44.9–92.2)	11/18	78.6 (49.2–95.3)	1	10/14	58.8 (32.9–81.6)	11/14	61.1 (35.7–82.7)	1

	LAM after α-mannosidase treatment of urine		LAM alone			LAM after α-mannosidase treatment of urine		LAM alone		
CD4 values < 200	Sensitivity (% (95% CI)		Sensitivity (% (95% CI)		*p* value^b^	Specificity (% (95% CI)		Specificity (% (95% CI)		*p* value^b^
	Positive/N		Positive/N			Negative/N		Negative/N		
Both Sites^a^	27/39	60 (44.3–74.3)	28/39	62.2 (46.5–76.2)	1	95/113	88.8 (81.2–94.1)	96/113	89.7 (82.3–94.8)	1
Site1	16/27	53.3 (34.3–71.7)	17/27	54.8 (36–72.7)	1	88/102	88.9 (81–94.3)	88/102	89.8 (82–95)	1
Site 2	11/12	73.3 (44.9–92.2)	11/12	78.6 (49.2–95.3)	1	7/11	87.5 (47.3–99.7)	8/11	88.9 (51.8–99.7)	1

^a^Site = Site 1 includes UAI 1 = “Dr. Isaac Cohen Alcahé” UAI and Rodolfo Robles Hospital. Site 2 includes UAI 2 = “Dr. Carlos Rodolfo Mejía” UAI and Roosevelt Hospital

^b^ Fischer exact Chi square test was used in comparisons among UAI

### TB incidence rates and mortality rates and risk factors

TB incidence and mortality rates in our cohort of PLWH with TB clinical symptoms were calculated using person-years (PYs). Overall, 59 PLWH were diagnosed with TB by the LAM-test, and 53 of those died. TB incidence was of 21.4/100 PYs with 95% CI of (16.6–27.6) for a total period of follow up of 275.6 PYs with a median time of follow up of 333 days and IQR (117–521). TB risk was of 16.3% with 95% CI of (12.9–20.5). To determine the mortality rate, 11 PLWH were excluded from analysis as their death dates were not reported; thus the 42 recorded deaths gave a mortality rate of 11.1/100 PYs with 95% CI (8.2–15.0) for a total period of follow up of 378.0 PYs, with a median time of follow up of 410 days and IQR (230–571). Moreover, 19/42 (45.2%) and 31/42 (73.8%) of our study participants died during the first 3 and 6 months, respectively, after enrolment. Mortality risk (n = 53) was of 14.9% with 95% CI of (11.6–19.1). Of the PLWH that died, 21/44 (47.7%) were taken second line cART, and 9/53 (17.0%) were not taken cART or their cART status was unknown.

### Risk factors as predictors of mortality with TB clinical symptoms

**Fig. 2:**
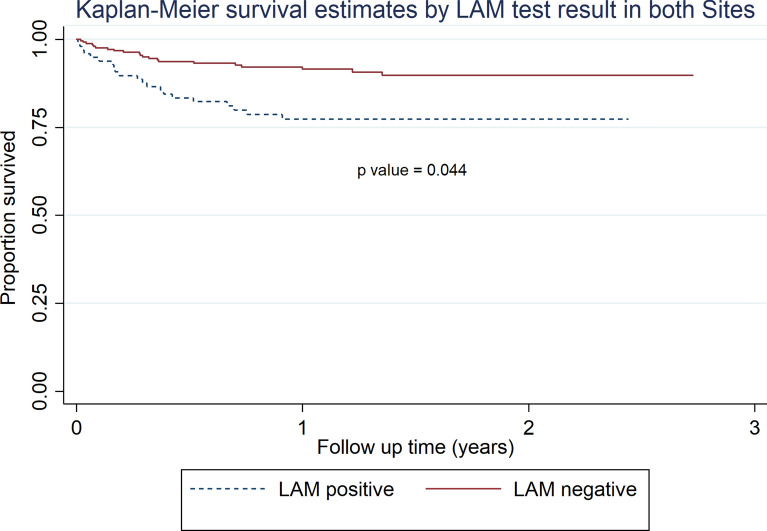
Kaplan–Meier survival estimates by LAM-test result

Results evaluating risk factors, as predictors for mortality and mortality outcomes for the 343 PLWH enrolled in this study, are shown in Table 3. After the follow up period, participants with a positive LAM-test result (with and without α-mannosidase treatment) had an aHR of death of 2.0 (1.0–3.8) *p* value = 0.044 as compared to PLWH with a negative LAM-test result. Overall, PLWH taking second line cART treatment had an aHR of death of 2.4 (1.1–5.0) *p* value = 0.023 compared to PLWH taking first line cART. Interestingly, specific for UAI 1, being female [aHR of 2.4 (1.41–5.4) *p* value = 0.03] and having a BMI < 17 kg/m^2^ [aHR of 2.7 (1.1–6.9) *p* value = 0.029] were also considered predictors of mortality. Kaplan–Meier survival estimates are presented in Fig. 2.

**Table 3:** Adjusted (aHR) Cox Hazard ratios for predictors of mortality in 343 PLWH with TB symptoms by Site

	Both Sites^a^		Site 1^a^		Site 2^a^	
	aHR	*p* value	aHR	*p* value	aHR	*p* value
Age^b^						
≤ 20	ref		ref		NA	NA
21–30	2.42 (0.29–20.17)	0.412	1.00 (0.12–8.43)	0.998	ref	
31–40	2.26(0.27–18.53)	0.448	0.63 (0.07–5.30)	0.673	1.01 (0.30–3.46)	0.983
41–50	2.33 (0.28–19.06)	0.43	1.08 (0.13–8.69)	0.943	0.71 (0.16–3.06)	0.643
51–60	2.72 (0.31–24.20)	0.368	0.23 (0.01–3.91)	0.307	2.20 (0.56–8.66)	0.257
>60	1.43 (0.13–15.66)	0.957	0.69 (0.07–6.32)	0.741	NA	NA
Sex						
Men	ref		ref		ref	
Women	1.31 (0.65–2.61)	0.448	0.51 (0.14–1.87)	0.312	2.43 (1.09–5.43)	0.03
Diabetes^c^						
No	ref		ref		ref	
Yes	0.67 (0.12–3.86)	0.658	NA	NA	0.52 (0.07–4.32)	0.543
BMI^d^						
≥ 17 kg/m^2^	ref		ref		ref	
< 17 kg/m^2^	1.41 (0.72– 2.79)	0.317	0.58 (0.12–2.73)	0.495	2.77 (1.11–6.92)	0.029
ART^e^						
1st line	ref		ref		ref	
2nd line	2.39 (1.12–5.07)	0.023	6.79 (2.16–21.31)	0.001	0.93 (0.42–2.05)	0.853
LAM						
Negative	ref		ref		ref	
Positive	1.98 (1.02–3.84)	0.044	1.08 (0.32–3.67)	0.897	2.10 (0.94–4.67)	0.069

*NA* Not applied due to sample size limitations

The following confounders were used for adjustment: Age, sex, CD4 values, cART, viral load values, and WHO HIV stage

^a^ Site = Site 1 includes UAI 1 = “Dr. Isaac Cohen Alcahé” UAI and Rodolfo Robles Hospital

Site 2 includes UAI 2 = “Dr. Carlos Rodolfo Mejía” UAI and Roosevelt Hospital

^b^ Age was used as a continuous variable in the multivariable Cox regression for “Dr. Carlos Rodolfo Mejía-Villatoro” UAI

^c^ Hba1c measurements in  % above 6.15 were considered the threshold for diabetic patients

^d^ In kg/m^2^

^e^1st and 2nd line regimens are shown in Table 1

## Discussion

Currently, this is the first reported diagnostic accuracy analysis of the urine LAM-test performed in Central America in PLWH. It is also the first time that an innovative procedure using α-mannosidase pre-treatment of urine was implemented in clinical settings to evaluate its diagnosis accuracy [14]. Importantly, a positive LAM-test result confirmed mortality prediction in our PLWH cohort with TB clinical symptoms as previously reported [23–28].

Our published laboratory data indicate that α-mannosidase treatment of LAM-spiked human urine using purified LAM from different *M. tuberculosis* strains, can increase tenfold the detection of LAM molecules in urine by the LAM-test [14]. Our field validation analysis of the LAM-test performed in α-mannosidase treated vs. untreated urine was run in two different clinical settings. On one hand, PLWH attending Site 1 used less second line cART, had higher CD4 T cell numbers and lower viral load values, and lower mortality rates compared to PLWH attending Site 2. Most of PLWH attending Site 1 were recruited as ambulatory patients, compared to a higher proportion of hospitalized patients recruited in Site 2, which includes the largest HIV clinic of the country located in Guatemala City. Differences in baseline characteristics of PLWH in these two Sites might explain some of the differences in diagnostic accuracy of the LAM-test. However, the LAM-test when compared to the composite reference gold standard performed equally in both Sites, regardless of the severity of WHO clinical stage, cART status, viral load, and CD4 T cell numbers. In addition, recent analysis show similar HIV cascades of care in both Site1 and Site 2 [29].

In this study, the LAM-test sensitivity in PLWH with < 50 CD4 T cells/µl was similar to PLWH with < 200 CD4 T cells/µl. A recent meta-analyses [26] showed a median pooled LAM-test sensitivity for TB diagnosis in PLWH of 45% with 95% credible interval of (29 to 63%), which is similar to our results in this study [(56.1% with 95% CI (43.3–68.3)] but with a smaller range of interval overlaps.

Contrary to our study in the laboratory setting, where α-mannosidase treatment of LAM spiked human urine significantly increased the efficiency of the LAM-test [14], we did not find any sensitivity and specificity differences comparing the LAM-test results in α-mannosidase treated vs. untreated urine using our composite gold referenced standard. This could be explained by several factors, including the viability and storage conditions of the α-mannosidase enzyme being used in the IAUs, as well as the use of a twofold diluted urine in sodium bicarbonate when performing the LAM-test in the α-mannosidase treated urine. This was performed with the rationale behind that sodium bicarbonate impairs micelle formation of LAM molecules in aqueous buffer [30, 31], but at the same time, the tested urine was diluted twofold, which could affect the sensitivity of the LAM-test. Another potential reason is the non-use of low-binding protein tubes during the test, as well as inconsistency in the incubation periods and temperature, showing in the lab setting to be optimal at 37 °C for 15 min [32].

Conversely, LAM in the infected host could be metabolized prior reaching the urine and thus, different epitopes could be exposed on LAM molecules making the α-mannosidase treatment less relevant than in the laboratory in vitro setting. However, in this regard ongoing studies show that this later possibility may not be plausible, as mannose-monocapped LAM motifs maybe detected in urine isolated from active TB patients [33].

Indeed, we found 3 PLWH that had an α-mannosidase LAM-test positive result, which were negative without this treatment. Of these, only 1 had a positive culture. Interestingly, there were 5 PLWH that had a LAM-test negative result only after α-mannosidase treatment, that otherwise were positive by the LAM-test using untreated urine. Of these, only 1 had a TB culture confirmed, indicating that α-mannosidase treatment of urine could potentially help in reducing false positive LAM-test results and detect true TB positives that otherwise could be missed. This is the first time that a modification of the LAM-test to increase its sensitivity is performed in real hospital-based settings worldwide showing promising results in its feasibility; however, further careful large-scale implementation and comparison analyses are necessary to verify its true usefulness in the field, especially in rural areas with limited TB diagnosis testing accessibility.

The impact of the current Coronavirus disease 2019 (COVID-19) pandemic on the number of new TB and HIV cases is unknown. Thus, in the years to come, improving TB diagnosis in PLWH is a key target to achieve towards the End TB strategy by 2030 [34–36]. TB/HIV services integration is highly recommended to improve TB and HIV treatment outcomes, as well as to establish TB/HIV programmatic indicators for surveillance, and monitoring and evaluation purposes [37, 38]. In this regard, TB incidence and mortality rates in PLWH with TB symptoms found in our PLWH cohort showed that even in HIV clinics with a “One Stop service delivery model” for TB and HIV care, TB incidence and mortality are still a serious concern. Our TB incidence results of 17.9/100 PYs and 95% CI of (10.4–30.8) in PLWH with < 100 CD4 T cells/µl, are similar to those of 25.5/100 PYs and 95% CI of (21.6–30.3) found in a PLWH cohort with similar CD4 T cell values in South Africa [39]. Indeed, our TB incidence rates of 22.5/100 PYs with 95% CI of (11.2–45.0) in PLWH with < 50 CD4 T cells/µl, are far higher than those of 4.2/100 PYs with 95% CI (1.4–12.7) found in a cohort of PLWH in Nigeria with similar CD4 T cell values [40]. Several studies have emphasized different factors behind high proportions of AIDS defining illnesses such as late presentation, TB diagnostic delay, and attrition in PLWH [27]. Social forces are key determinants in explaining TB morbidity and mortality outcomes in the Central American region that need to be further explored.

Our data also indicate that a positive LAM-test result in PLWH with TB clinical symptoms predicts mortality outcomes [aHR of 1.98 (1.0–3.8) *p* value = 0.044]. This was previously suggested in recent sub-Saharan Africa studies involving hospital inpatients [4, 41], as well as in PLWH receiving treatment for HIV-associated active TB in sub-Saharan Africa and Thailand [42, 43], and in newly diagnosed PLWH screened for TB in South-Africa [44].

## Limitations

This pilot study was originally focused as a feasibility study and a proper sample size calculation is missing. LAM-test results using two independent readers in diagnostic accuracy analysis of the composite gold standard vs. the LAM-test (urine untreated vs. α-mannosidase treated) were not properly recorded. Thus, Kappa concordance agreement studies were not performed. LAM-test after urine α-mannosidase treatment was performed in twofold diluted urine in sodium bicarbonate in place of being directly performed in undiluted urine. However, LAM-test using α-mannosidase diluted samples performed equally to LAM-test alone with undiluted samples Finally, LAM-test band intensity was not properly registered and thus, results were only ascertained as positive or negative using the LAM-tests grade 1 to 4 independently of the intensity of the band visualized, which limited to know if α-mannosidase treatment, a part of uncovering true positives, was able to allow better detection of LAM molecules in urine by the LAM-test, and further correlate the bacterial burden (culture) with the intensity of the LAM-test band (i.e., a direct correlation between more bacteria producing more LAM molecules resulting in a high intensity band).

## Conclusions

In this study, α-mannosidase treatment of urine did not significantly increase the LAM-test performance; however, the performance of the LAM-test after α-mannosidase treatment of urine needs to be carefully revaluated in the field at larger scale and proper controls. This simple α-mannosidase treatment of urine could increase the sensitivity of the LAM-test (and other urine-based test such the new FujiLAM [45]), when compared to untreated urine in field settings using large cohorts of HIV-uninfected and PLWH clinically suspicious of active TB disease. In this regard, it will be important to evaluate in the field, if α-mannosidase treatment of urine can certainly increase the detection of LAM molecules and correlate these results with bacterial burden. Finally, high rates of TB incidence and mortality were found within established “One Stop” services, and a positive LAM-test result could predict mortality in PLWH with TB disease clinical symptoms.

## Data Availability

Due to the ethically sensitive nature of this research, datasets generated during the current study are not publicly available but available to researchers only without personal identifiers.
