# Correction to: Accuracy of the tuberculosis point-of-care Alere determine lipoarabinomannan antigen diagnostic test using α-mannosidase treated and untreated urine in a cohort of people living with HIV in Guatemala

**DOI:** 10.1186/s12981-022-00466-z

**Published:** 2022-10-20

**Authors:** Juan Ignacio García, Johanna Meléndez, Rosa Álvarez, Carlos Mejía-Chew, Holden V. Kelley, Sabeen Sidiki, Alejandra Castillo, Claudia Mazariegos, Cesar López-Téllez, Diana Forno, Nancy Ayala, Joan-Miquel Balada-Llasat, Carlos Rodolfo Mejía-Villatoro, Shu-Hua Wang, Jordi B. Torrelles, Janet Ikeda

**Affiliations:** 1 Fundació Sida I Societat, Technical Advisor Unit (UAT), Escuintla National Hospital, Escuintla, 5001 Guatemala; 2 Tuberculosis Group, Population Health Program, Texas Biomedical Research Institute, San Antonio, TX 78227 USA; 3 Unidad de Atención Integral del VIH E Infecciones Crónicas del Hospital Roosevelt “Dr. Carlos Rodolfo Mejía Villatoro”, Guatemala City, Guatemala; 4 Sección de Microbiología, Departamento de Laboratorios Clínicos, Hospital Roosevelt, Guatemala City, Guatemala; 5 Division of Infectious Diseases, Department of Medicine, Washington University School of Medicine, St. Louis, MO 63110 USA; 6 Clinica de Atención Integral Dr. Isaac Cohen Alcahé, Hospital de Especialidad Dr. Robles, Quetzaltenango, Guatemala; 7 Asociación de Investigación, Desarrollo Y Educación Integral (IDEI), Quetzaltenango, Guatemala; 8 Division of Global HIV/AIDS, Centers for Disease Control and Prevention, Central America Regional Office, Guatemala City, Guatemala; 9 National Reference Laboratory, Amatitlan, Guatemala; 10 Department of Pathology, The Ohio State University, Wexner Medical Center, Columbus, OH USA; 11 Internal Medicine Department, Infectious Disease Division College of Medicine (COM), The Ohio State University (OSU), Columbus, OH 43210 USA

## Correction to: AIDS Res Ther (2020) 17:62 https://doi.org/10.1186/s12981-020-00318-8

In the original publication of this article [1], the authors were notified that there were some errors in Table 2 that were also present in the main text, specifically in the results section of the abstract, the overall sensitivity of the LAM-test of 56.1% with 95% CI of (43.3–68.3) should have been 62.7 with 95% CI of (49.1–75); the LAM-test sensitivity in PLWH with < 200 CD4 T cells/µl of 62.2% (95% CI 46.5–76.2) should have been of 71.8% with 95% CI of (55.1–85), and the differences in sensitivity when comparing LAM-test results obtained from untreated vs. α-mannosidase treated urine had been deleted.

The sentence “the two participating UAIs using the Fischer exact test” in the statistical analysis sub-heading should have been “the LAM tests using the McNemar exact test”.

In the results section, the LAM-test sensitivity of 56.1% with 95% CI of (43.3–68.3) should have been 62.7 with 95% CI of (49.1–75), and the LAM-test sensitivity in PLWH with < 200 CD4 T cells/µl of 62.2% (95% CI 46.5–76.2) should have been of 71.8% with 95% CI of (55.1–85), the acronym “UIA” should have been “UAI”; the sentence “the defined gold standard composite” should have been “the defined reference standard”; the word “gold” has been eliminated; the sentence “composite gold referenced standard” should have been “composite reference standard”; the acronym “IAUs” should have been “UAI”, and the term “composite gold standard” should have been “composite reference standard”.

As a result, the authors have corrected Table 2. All sensitivity and specificity values have been corrected; the McNemar exact test has replaced the Fischer exact test for p value calculations, and these p values have been corrected; the footnote has been modified to reflect these changes.

**Table 2:**
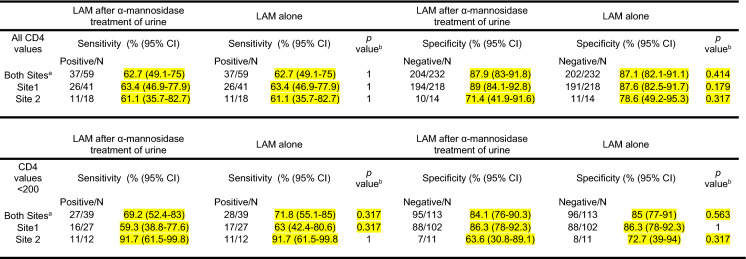
Sensitivity and specificity comparisons of LAM test after α-mannosidase treatment of urine vs. LAM test by Site and CD4 values using a composite reference standard

^a^Site = Site 1 includes UAI 1 = "Dr. Isaac Cohen Alcahé" UAI and Rodolfo Robles Hospital. Site 2 includes UAI 2 = "Dr. Carlos Rodolfo Mejía" UAI and Roosevelt Hospital

^b^McNemar exact test was used when comparing LAM test alone and LAM test after α-mannosidase treatment

The authors apologize for any inconvenience.
